# Bioremoval of humic acid from water by white rot fungi: exploring the removal mechanisms

**DOI:** 10.1186/s13568-016-0293-x

**Published:** 2016-11-22

**Authors:** M. Zahmatkesh, H. Spanjers, M. J. Toran, P. Blánquez, J. B. van Lier

**Affiliations:** 1 Department of Water Management, Section Sanitary Engineering, Delft University of Technology, PO Box 5048, 2600 GA Delft, The Netherlands; 2 Chemical, Biological and Environmental Engineering Department, Escola d’Enginyeria, Universitat Autònoma de Barcelona, Bellaterra, Barcelona, Spain

**Keywords:** White rot fungi, Humic acid, Biodegradation, Biosorption, Laccase, Manganese peroxidase, Cytochrome P450

## Abstract

Twelve white rot fungi (WRF) strains were screened on agar plates for their ability to bleach humic acid (HA). Four fungal strains were selected and tested in liquid media for removal of HA. Bioremediation was investigated by HA color removal and changes in the concentration and molecular size distribution of HA by size exclusion chromatography. *Trametes versicolor* and *Phanerochaete chrysosporium* showed the highest HA removal efficiency, reaching about 80%. Laccase and manganese peroxidase were measured as extracellular enzymes and their relation to the HA removal by WRF was investigated. Results indicated that nitrogen limitation could enhance the WRF extracellular enzyme activity, but did not necessarily increase the HA removal by WRF. The mechanism of bioremediation by WRF was shown to involve biosorption of HA by fungal biomass and degradation of HA to smaller molecules. Also, contradicting previous reports, it was shown that the decolorization of HA by WRF could not necessarily be interpreted as degradation of HA. Biosorption experiments revealed that HA removal by fungal biomass is dependent not only on the amount of biomass as the sorbent, but also on the fungal species. The involvement of cytochrome P450 (CYP) enzymes was confirmed by comparing the HA removal capability of fungi with and without the presence of a CYP inhibitor. The ability of purified laccase from WRF to solely degrade HA was proven and the importance of mediators was also demonstrated.

## Introduction

### Humic substances

Humic substances are the most widespread natural organic substances that are ubiquitous in the environment, both aquatic and terrestrial. They are found in sediments, peat, lignites, brown coal, sewage, composts and other deposits (Stevenson 1995; Hedges et al. 2000). Humic substances are not well defined, but are generally divided into three fractions based on their solubility in acids and alkalis: humic acid (HA) that is soluble in alkali and insoluble in acid; fulvic acid (FA) that is soluble in alkali and acid, and humin that is insoluble in both alkali and acid. Humic substances are comprised mainly of aromatic, aliphatic, phenolic and quinonic components, which are covalently bound through C–C, C–O–C and N–C bonds (Stevenson 1995). In nature, humic substances are extremely resistant to biodegradation (Paul et al. 1997). HA can absorb heavy metals and xenobiotic compounds, hence increase their solubility and mobility in water. Also during chlorination of treated water, HA can cause the formation of trihalomethanes and other carcinogenic and mutagenic substances. Therefore their presence in industrial effluents can cause damage to the ecosystem. (Morimoto and Koizumi 1983; Qi et al. 2004). Furthermore, the presence of humic compounds results in colored effluents leading to esthetic constraints when these effluents are discharged to the environment (Saar and Weber 1980; Yang and Shang 2004).

Microorganisms are the driving force behind the formation, transformation, degradation and mineralization of humic substances. Although bacteria dominate the environment and participate in turnover of humic substances (Paul et al. 1997; Wang et al. 2013), their ability to degrade stable macromolecules such as HA is limited (Filip and Tesařová 2004).

### White rot fungi

White rot fungi (WRF) are the most abundant wood degraders in nature, which possess the unique ability of efficiently degrading lignin to CO_2_ (Hataka 2005; Abdel-Hamid et al. 2013). They are also able to decompose several aromatic pollutants or xenobiotics and thus can be used in (waste) water treatment (Pinedo-Rivilla et al. 2009; Nguyen et al. 2014). The non-specific nature of WRF enzymes has been reported to be the key factor in their ability to degrade some complex aromatic polymers with molecular structures similar to lignin (Tišma et al. 2010; Mendonça Maciel et al. 2010). Extracellular Peroxidases and laccase have been reported to be the key enzymes involved in the degradation of recalcitrant aromatic polymers by WRF (Mester and Tien 2000; Zahmatkesh et al. 2010; Mendonça Maciel et al. 2010). Cytochrome P450 (CYP) enzymes comprise another group of enzymes of which their involvement in the degradation of some organic compounds by WRF has been demonstrated (Ning and Wang 2012; Kelly and Kelly 2013; Zhang et al. 2015). CYPs are membrane-bound hemoproteins that catalyze hydroxylation, epoxidation and monooxygenation reactions (Neve and Ingelman-Sundberg 2008; Aranda 2016). In fungi, CYPs are involved in the biosynthesis of secondary metabolites and ergosterol, and also in catabolic reactions that lead to the degradation of xenobiotic compounds (Aranda 2016).

### Ambiguities and knowledge gaps

The removal of HA by WRF has been studied before, and It was shown that WRF can degrade humic acid (Ralph and Catcheside 1997; Grinhut et al. 2011a). However, there are still ambiguities regarding the level of biosorption and degradation/transformation of HA by WRF. The decolorization of an HA solution has been accepted as an indication of the decrease in HA concentration in that solution (Stevenson 1995), which could be due to degradation, conversion or sorption of HA. Yet the decolorization has also been used to estimate the degradation of HA (Grinhut et al. 2011a, b). The ability of laccase to degrade HA has been studied before. However, results are not in agreement. Fakoussa et al. (1999) and Steffen et al. (2002) observed a correlation between laccase activity and the degradation of HA in a WRF culture and concluded that laccase is involved in the degradation of HA by WRF. On the contrary, Zavarzina et al. (2004) reported that laccase could polymerize HA. They also reported that the degradation of HA by WRF’s laccase could result in an increase in color, whereas polymerization of HA was leading to a decrease in the color. Besides, it was reported that HA have an inhibitory effect on laccase activity, which is in contrast with results that show a stimulation of laccase activity in the presence of HA (Willmann and Fakoussa 1997).

In this study, the ability of 12 different strains of WRF to remove HA from solid and liquid media was investigated. HA color removal was compared with changes in concentration and molecular size distribution measured by size exclusion chromatography (SEC) to clarify the ambiguity about the interpretation of decolorization of HA, whether it is indicating degradation of HA or only decrease in its concentration. The SEC results were analyzed numerically and the reliability of average molecular weight (calculated based on the SEC results) to conclude the degradation of HA was investigated. The correlation between the SEC results and color measurement was studied to clarify whether the degradation of HA result in a decrease or an increase in the color. Mechanism of HA removal by WRF was explained by distinguishing the sorped HA from degraded/transformed HA. The extracellular enzyme activities were measured, and the relationship between extracellular enzyme activities of WRF and HA removal was investigated. The ability of laccase to degrade HA was studied using pure laccase, and also the effect of different mediators on the performance of laccase was tested. Additionally, the contribution of cytochrome enzymes to HA removal by WRF was studied.

## Materials and methods

### Fungal strains and chemicals

Twelve WRF strains (Table 1) were obtained from the fungal stock culture collection of Wageningen University and Research (Wageningen, the Netherlands) and DSMZ (Germany). The fungal strains were pre-cultivated on 2% malt extract agar, and subcultures were made periodically every 40 days to keep the cultures fresh. All the chemicals including coal humic acid powder and pure fungal laccase (from *Trametes versicolor*) were purchased from Sigma-Aldrich (Germany), unless otherwise stated.

**Table 1:** Qualitative results of pre-screening on agar plates

Fungal strain	Growth		Bleaching	
	Media A	Media B	Media A	Media B
*Clitocybula dusenni MES11937*	0	1	0	0
*Trametes suaveolens MES12281*	0	2	0	0
*Trametes suaveolens MES11922*	0	1	0	1
*Trametes versicolor DSMZ 3086*	0	4	0	3
*Trametes versicolor MES02055*	0	2	0	1
*Ceriporiopsis subvermispora MES13094*	0	1	0	1
*Pleurotus sajor*-*caju MES03464*	0	4	0	2
*Pleurotus ostreatus MES00036*	0	4	0	2
*Pleurotus ostreatus MES00050*	0	3	0	1
*Pleurotus ostreatus MES01475*	0	1	0	1
*Pleurotus ostreatus MES03772*	0	3	0	1
*Phanerochaete chrysosporium DSMZ 1556*	0	3	0	2

Media “A”: HA as sole carbon source, Media “B”: with additional carbon source (PDB)

In the media type “A”, using HA as the sole carbon source, no significant growth was observed for any of the WRF strains. In media type “B” on the other hand, growth was observed. This shows that WRF strains could not utilize HA as the sole carbon source and needed additional carbon sources to grow

### Media

#### Preparation of HA stock solution

Humic acid powder (4 g) was dissolved in 200 mL of NaOH solution (0.1 M) and mixed for 30 min. The solution was centrifuged (7000 rpm, 20 min) to remove the particulates. Then 100 mL of phthalate buffer (0.5 M) was added to the particulate-free HA solution, and pH was adjusted to 4.5 with HCl. The buffered solution was centrifuged again (7000 rpm, 20 min) and the supernatant was used as the HA stock solution. For each set of experiments a fresh batch of the stock solution was prepared. The concentration of humics in the stock solution was determined by drying (48 h, 100 °C) 30 mL of the stock solution (triplicate) and deducting the weight of the buffer from the dry weight. The humics concentration of the stock solution was 8 g/L (±0.4 g/L). For all the experiments HA stock solution was filtered (0.45 μm) prior to use.

#### Liquid media

Liquid media was adapted and simplified from the defined culture media for growth and enzyme production of WRF as described by Kirk et al. (Kirk et al. 1978). Defined media was prepared as defined nitrogen limited (NL) and nitrogen sufficient (NS) media including basal media, minerals (trace elements) and vitamins along with carbon and nitrogen sources. In all cases, media contained glucose as carbon source (56 mM) unless otherwise stated. Ammonium tartrate was used as the nitrogen source in a final concentration of 2 mM for NL media and 20 mM for NS media. Media was supplemented with sodium phthalate buffer (10 mM, pH 4.5). The defined media were spiked (50 mL/L) with HA from HA stock solution when needed (final concentration of HA in media: ~400 mg/L).

#### Solid media

Two types of media were prepared to evaluate the ability of the WRF to grow in the presence of HA. The media Type “A” contained only HA (~250 mg/L) as the carbon source, along with the other elements of NS medium. Media Type “B” was basically the Kirk medium (NS) with glucose as the carbon source, dosed with HA (~250 mg/L). Both media were supplemented with 10 g/L Agar for solidification.

### Experimental procedures

#### Pre-screening on agar plates

Plastic petri dishes (90 mm inner diameter) containing about 35–40 mL solid media were used in pre-screening experiments. All petri dishes were inoculated with an agar piece (5 × 5 mm) of the respective pre-cultivated fungi. Plates were made in triplicates for each media type and fungal strain and incubated at 25 °C for 15 days in the dark. The fungal growth and decolorization of HA were both estimated qualitatively by observing the diameter and density of the mycelia on the agar plate and the bleaching of the agar medium.

#### HA removal by fungi from water

Selected fungal strains from the pre-screening experiment were used in the defined NL and NS liquid media. Experiments were done in 500 mL flasks filled with 150 mL defined media, inoculated with five pieces of fungal agar. Bioremediation flasks were prepared in quadruplicate, two of which were subjected to sampling during the incubation period for the analysis. The other two were kept intact and only used at the end of the incubation for the recovery process. Flasks were closed with cotton stoppers and incubated in a shaker incubator (26 ± 1 °C, 150 rpm).

Three different sets of controls were prepared to ensure that the observations of the experiments were linked to the fungi. The first set of controls were uninoculated NL and NS media supplemented with HA (uninoculated controls), to reveal any chemical interaction between the defined media and HA, verifying the stability of HA in the media. The second set of controls was NL and NS media inoculated with the respective fungal strains, in the absence of HA (HA-free control). This was done to monitor any changes in the color of the media as a result of the fungal growth, as well as a possible production of metabolites that can interfere with HA analysis via SEC. The third control was uninoculated HA solution without defined media, to test the stability of HA solution itself with regards to color and MW distribution.

#### Recovery of sorped HA from fungal mycelia

To recover the sorped HA from mycelia, a weighted amount of NaOH was added to each jar to a final concentration of 1 M (pH > 12), and then the fungal mycelia were disrupted by means of vigorous mixing for 2 h, followed by 2 min of sonication. At the end, samples were withdrawn and filtered through 0.45 μm filters following the procedure of Ralph and Catcheside (1994, 1996).

#### HA sorption by deactivated fungal mycelia

In order to investigate the capability of fungal mycelia for biosorption of HA, four fungal strains were grown separately in potato dextrose broth (PDB) in a shaker incubator for 10 days (26 ± 1 °C, 150 rpm). Fungal biomass, grown in the form of pellets, were collected and washed five times with distilled water. Afterwards the jars containing the fungal pellets in distilled water were autoclaved (121 °C, 20 min) to deactivate the fungi. The deactivated (heat-killed) fungal pellets were then used for the biosorption experiment. Different amounts of biomass for each fungal strain were used to study the HA sorption on fungal biomass. The fungal biomass was transferred to 4 flasks containing 100 mL HA solution (~200 mg/L, pH 5) in four levels. The same amount of pellets were also filtered and dried (100 °C, 24 h) and weighted separately to estimate the dry biomass weight (DBW) of each experiment. Flasks were kept in a shaker incubator for 48 h (26 ± 1 °C, 130 rpm). At the end of the incubation period, 1 ml samples were withdrawn from each flask to be analyzed for color. Then the HA was recovered to estimate the efficiency of the recovery procedure via SEC analysis. Experiments were done in duplicate under sterile conditions.

#### HA degradation by purified laccase

The degradation of HA by laccase was performed in triplicate in 150 mL flasks containing 50 mL of laccase solution at 500 U/L in malonate buffer (pH 4.5). The effect of mediators was investigated by the addition of 1-hydroxybenzotriazol hydrate (HOBt), 2,2-azino-bis-(3-ethylbenzthiazoline-6-sulfonic acid (ABTS) and violuric acid (VA) at the final concentration of 1 mM (Mir-Tutusaus et al. 2014). Flasks were spiked with 2 mL of HA stock solution and kept in a shaker incubator for 48 h (26 ± 1 °C, 130 rpm) under sterile conditions. 2 ml samples at designated times were withdrawn and analyzed by SEC.

#### Cytochrome P450 inhibition

To study the involvement of CYP enzymes, the fungal pellets were incubated in the presence of 1-aminobenzotriazole (ABT) as the CYP enzymes inhibitor to hinder the activity of these enzymes (Marco-Urrea et al. 2006). Fungal pellets (~8 g wet weight) were incubated in NL and NS media (100 mL flasks containing 25 mL media) for 7 days and 1 mL samples were withdrawn at designated times for analysis. All flasks were prepared in triplicate.

### Analytical methods

#### Size exclusion chromatography (SEC)

Samples for SEC analysis were prepared by separating humic acid- like molecules from the media. Each sample (2 ml) was acidified (pH < 2) by adding 20 μl HCl (37%), and centrifuged (14,000, 20 min). The acid supernatant was separated as FA, and the precipitants were re-suspended by adding 2 mL NaOH (0.1 M) and used as HA portion of the sample (Hofrichter and Fritsche 1996). Both FA and HA portions of the samples were analyzed by SEC. The SEC was conducted using Phenomenex column (Yarra™ 3 µm SEC-2000, LC Column 300 × 7.8 mm, Ea) connected to an ultra fast liquid chromatography (UFLC) system (Shimadzu, Prominence) to detect changes in the concentration and MW of HA (and FA) molecules during the incubation with WRF. The method was adapted from a protocol that has already been developed for molecular size fractionation of HA (Asakawa et al. 2011), with slight modification. The mobile phase was 25% acetonitrile in ultra pure water supplemented with 10 mM sodium phosphate buffer (pH 7). The flow rate of the mobile phase was 1 ml/min and the injection volume was 10 μl. Polystyrene sulfonate standards (Polymer Standard Service, Germany) were used for the calibration of the column. Separation was done at 25 °C for 16 min and eluted substances were detected at 254 nm.

#### Other analysis

Decolorization of HA was assessed by measuring light absorbance at 450 nm (Hofrichter and Fritsche 1997).

Extracellular enzyme activities were determined spectrophotometrically in culture supernatant obtained by filtering through 0.45 μm syringe filters. Lignin peroxidase (LiP) was assessed at 30 °C using veratryI alcohol as substrate (Tien and Kirk 1988). MnP activity was assayed using Mn(II) as the substrate (Paszczyński et al. 1988). Laccase activity was measured by monitoring the oxidation of 2,6-dimethoxiphenol (DMP) as described before (Cruz-Morató et al. 2013). The enzyme activities were expressed in units (U: micromoles/min).

Fungal growth was assessed by measuring the dry biomass weight (DBW) of the fungal mycelia. The fungal biomass was harvested through filtration (10 μm paper filters, pre-weighted) and dried in pre-weighted aluminum cups (100 °C, 48 h). The net weight of the cups with and without the fungal biomass was calculated as the DBW.

## Results

### Pre-screening on agar plates

**Fig. 1:**
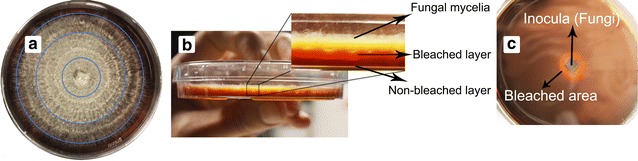
**a** Fungal growth on the humic-agar plate. **b** Vertical bleaching of humic acid agar media by WRF, **c** horizontal bleaching

A total of 12 WRF strains were tested during the pre-screening experiment using HA agar medium, with and without the additional carbon source. The fungal growth on the media was evaluated qualitatively based on observation of the diameter and density of growth around the inocula. Bleaching was also evaluated qualitatively by observing the horizontal and vertical bleaching of HA agar around the inocula. The typical growth of fungi in agar plate containing humic acid as well as bleaching of HA agar is shown in Fig. 1.

The results of the growth of the WRF strains and their bleaching effects on HA-agar medium are presented in Table 1. The levels of growth and bleaching are specified with numbers, from 0 for no growth (no bleaching) to 4 for excessive growth (bleaching).

Based on growth and bleaching results of the pre-screening experiments (Table 1), four strains of WRF, *Pleurotus sajor*-*caju MES03464, Pleurotus ostreatus MES00036, T. versicolor DSMZ 3086* and *Phanerochaete chrysosporium DSMZ 1556* were selected for screening in the liquid phase.

### Screening in liquid media (water)

**Fig. 2:**
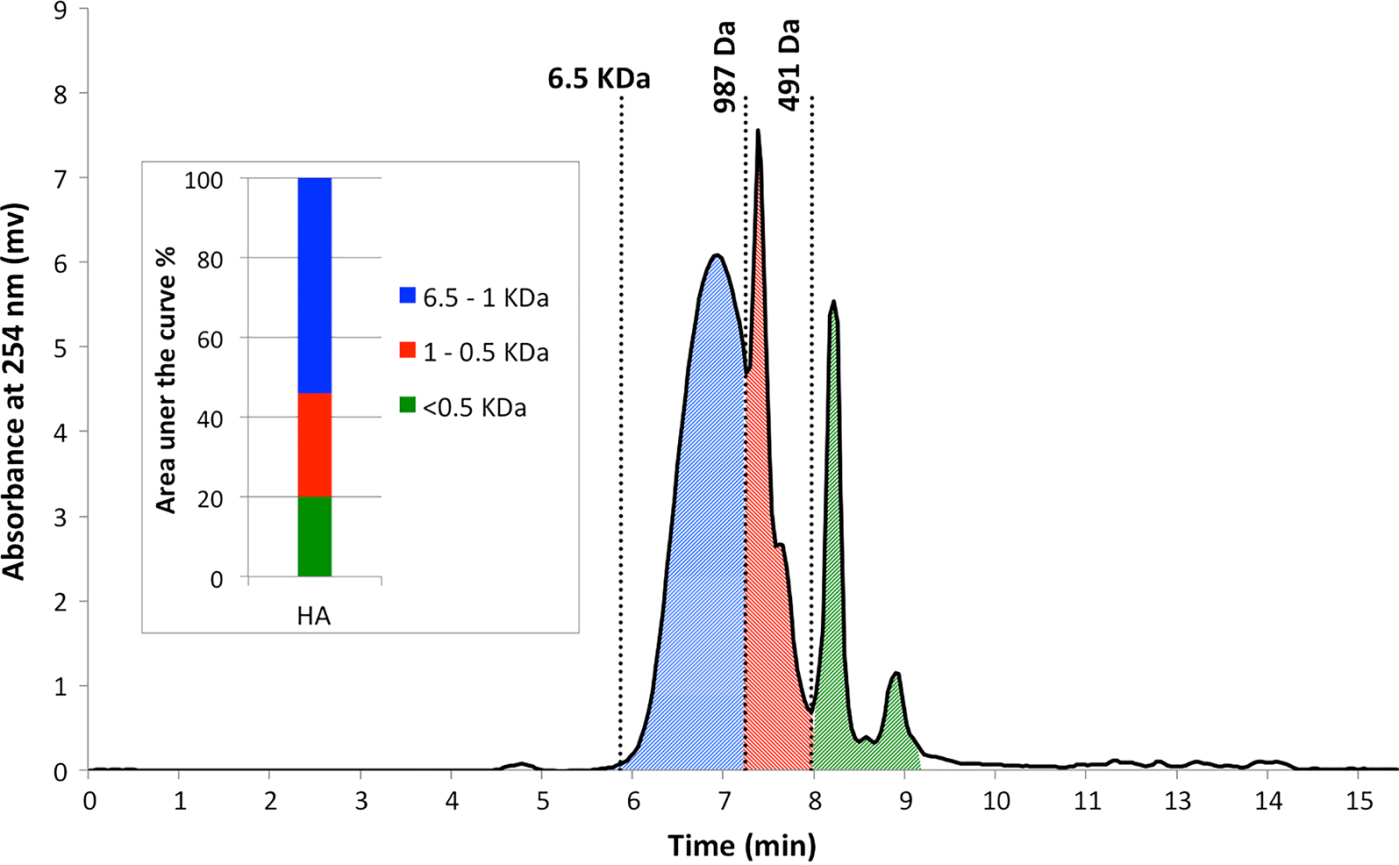
SEC chromatogram of HA and its stacked column presentation

The SEC analysis of the uninoculated HA solution showed that HA is a complex of molecules with a broad range of MW from 6.5 kDa to less than 0.5 kDa. In order to facilitate the comparison of SEC results during the fungal treatment experiments, each SEC chromatogram was sliced into three separate areas based on the main peaks, as it is shown in Fig. 2. The large (high molecular weight) HA molecules, having the molecular weight of 1–6.5 kDa (blue), comprise most (54%) of the HA complex. The medium size HA and building blocks, weighing between 1 and 0.5 kDa (red) cover around 26%, and small (low molecular size) HA weighing less than 0.5 kDa (green) cover the rest of the HA complex (20%). The total height of HA column (Fig. 2) representing the total area under the curve of HA chromatogram, can be used to qualitatively monitor variations in the concentration of HA. Also changes in the ratio between different portions of the HA (blue, red and green areas) indicate changes in the MW distribution of the HA complex. The average MW of HA was 1.41 kDa (±0.05). The FA portion of the HA solution was also subjected to SEC analysis. Although the HA solution was made using HA powder, still a relatively narrow peak was observed in the SEC chromatogram of the acid soluble portion of the HA solution (data not shown). The average MW of these fulvic-like molecules was 0.2 kDa (±0.03).

**Fig. 3:**
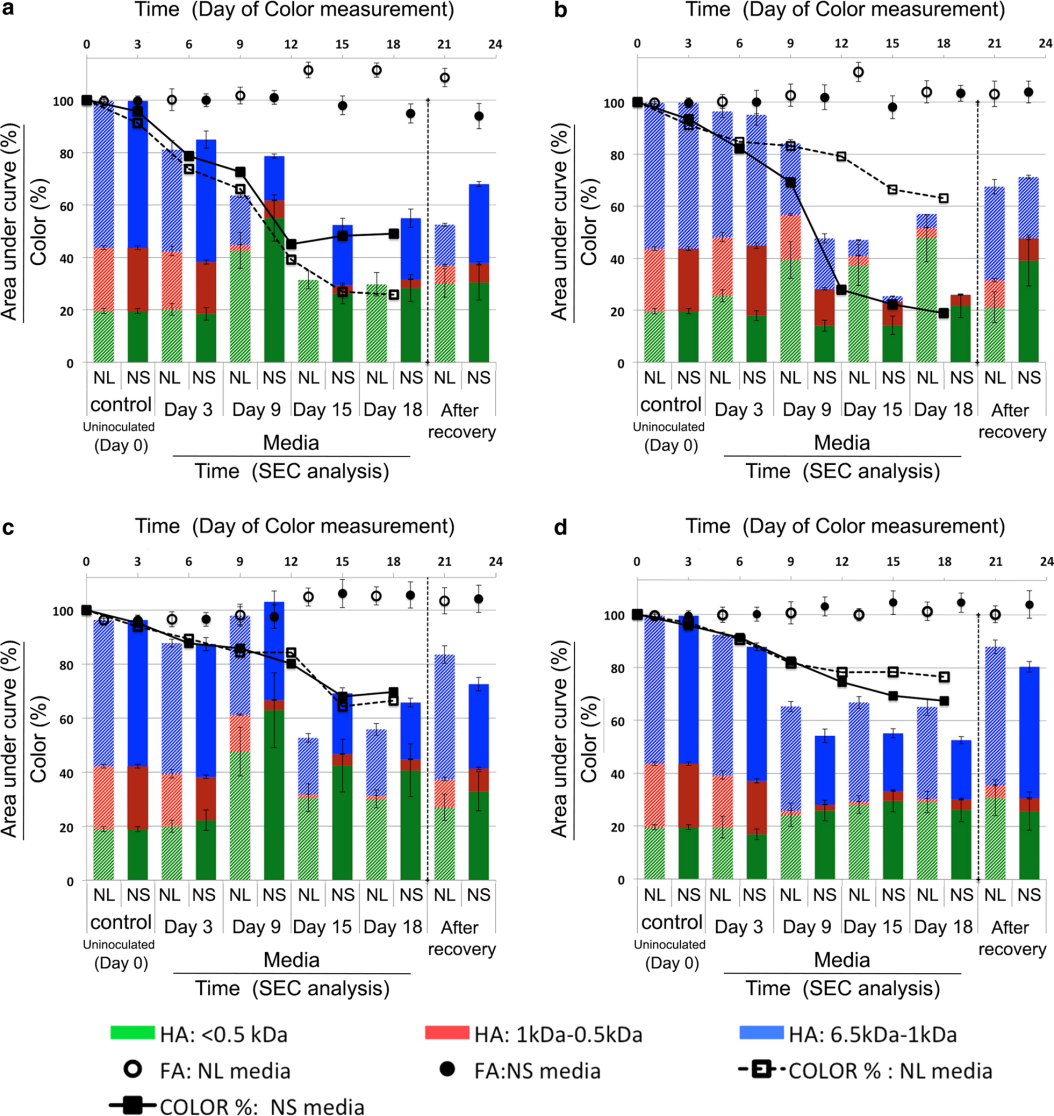
Decolorization of HA by four fungal strains and SEC results (area under the curve) of HA and FA content of the media. **a** *T. versicolor*, **b** *P. chrysosporium*, **c** *P. sajor*-*caju*, **d** *P. ostreatus*

The results of the HA color removal along with the results of the SEC analysis are shown in Fig. 3. The presented results of color measurement in Fig. 3 were corrected for the HA-free controls. Performing the recovery procedure on HA-free controls revealed that the color of fungal media even in the absence of HA, increased significantly during the recovery process (data not shown). Therefore it was not reasonable to use color as an indicator of HA concentration after the recovery process. The SEC results of the HA-free controls showed that no metabolites were produced during the incubation nor during the recovery process, that can interfere significantly with the SEC analysis of HA. Therefore, the SEC results can be used for analysis of HA during the incubation and after the recovery procedure. The uninoculated controls did not show any significant change in the color (<5%) nor in MW distribution, indicating the high stability of the HA solution.

*Trametes versicolor* and *P. chrysosporium* had the largest bleaching effect on the HA media of about 80% color removal. *P. chrysosporium* removed 80% of the color after 18 days of incubation in the NS media, and 40% in the NL media. *T. versicolor* showed a different behavior, removing 50% of the color after 18 days in NS media and 75–80% in NL media.

SEC results clearly indicated that *P. chrysosporium* could completely remove the large HA (blue) after 18 days of incubation in NS media. The removal of large HA by *T. versicolor* was incomplete in the NS media, although complete removal of large and medium size HA was achieved in the NL media. Looking at the different portions of humic acids (blue, red and green areas) after 18 days, for all the experiments except *P. chrysosporium* in NL media, a decrease in the concentration of large HA molecules (blue zones) coincided with an increase in the concentration of smaller HA molecules (red or green zones), suggesting incomplete degradation of large HA molecules to smaller HA molecules.

The changes in the concentration of FA were less significant than for HA molecules. For *T. versicolor*, 15% increase in FA concentration was observed after 18 days of incubation in NL media. However, in NS media, the FA concentration slightly decreased. *P. chrysosporium* and *Pleurotus* species showed a slight increase in FA concentration (5–10%). The correlation between the HA removal and increase in FA concentration suggests the conversion of HA molecules to FA molecules, which is in line with some previous reports(Hofrichter and Fritsche 1996).

The results of the SEC analysis after desorption of HA from fungal mycelia (recovery) are most important to understand the involvement of biosorption in the removal of HA by WRF. In case of *T. versicolor* in NL media, after the recovery (desorption), almost 50% of the initial HA was detected in the media via SEC, which means that from the 70% reduction in HA concentration after 18 days, at least 20% was due to the biosorption of HA molecules to fungal mycelia. It is obvious that for both NL and NS media, the ratio between the three portions of HA (blue, red, green areas) was shifted towards the smaller molecules (Fig. 3a, after recovery). The MW distribution was changed from 54: 26: 20% (large: medium: small) to (when normalized to 100%) 29: 13: 58% for NL media and 44: 11: 45% for NS media. The HA average MW after the recovery was reduced from 1.41 to 0.8 kDa in the NL media and 1.1 kDa in the NS media. Another important observation is the total area under the SEC curve, as an indication of HA concentration. In the culture of *P. chrysosporium*, the ratio between the three different portions of HA after recovery in NL media was 53: 16: 31% (large: medium: small) and in NS media it was 33: 12: 55%. Interestingly, the ratio (MW distribution) in the NL media (after the recovery) is close to the initial composition of HA, although about 30% degradation/transformation of HA was observed from total area under the curve. The average MW of HA in the NL media was 1.33 kDa and in the NS media it was 0.9 kDa. The results of the SEC analysis of *P. ostreatus* in the NS media shows that after fungal treatment (Fig. 3d, after recovery) the HA was comprised of 62% large HA (blue), 6% medium size (red) and 32% small HA substances (green). The average MW of the HA was 1.49 kDa, which indicates polymerization of HA when compared to the initial average MW of (1.41 kDa). Although from the total area under the SEC curve (height of the column), it is apparent that around 20% of HA were either degraded to smaller non-aromatic molecules or degraded/transformed to FA. Therefore monitoring the average MW of the HA complex is not a reliable way to draw conclusions about depolymerization or polymerization of HA. The reason is that depolymerization of HA could result in non-aromatic products or FA-like substances, which obviously will not be detected during the SEC analysis of HA.

The recovery results did not show any significant increase in the FA concentrations. This suggests that FA molecules have low affinity to biosorption by fungal mycelia.

As it can be seen in Fig. 3, there is a correlation between the total area under the HA chromatogram (height of the column) and the color of the media during the incubation period, which confirms the reliability of color measurement to monitor the HA concentration in the media during the incubation period.

Extracellular enzyme activities of the tested WRF strains were measured to investigate the possible role of these enzymes in the degradation of the HA molecules.

**Fig. 4:**
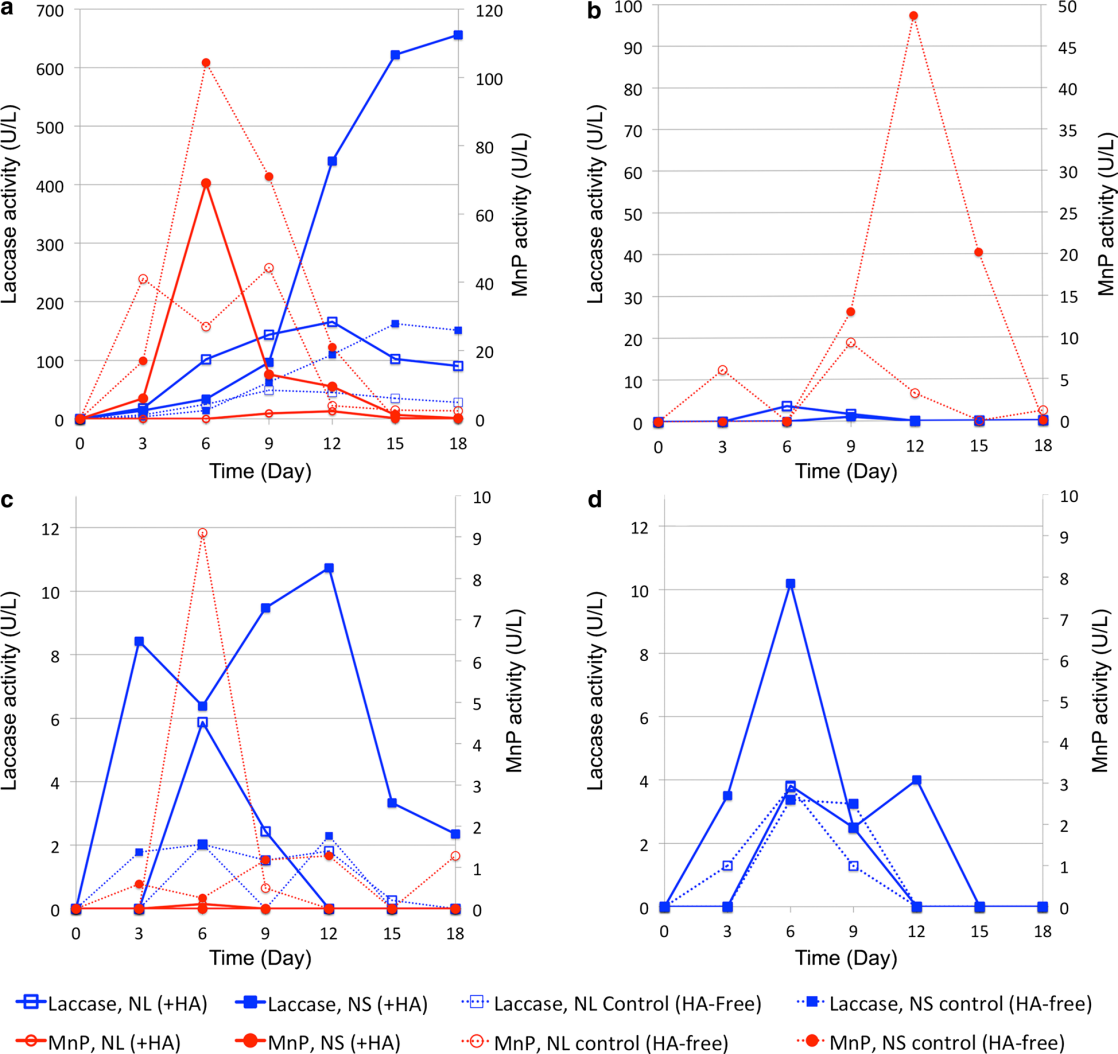
Extracellular enzymes activities of four tested fungal strains in NL and NS media with and without the presence of HA. **a** *T. versicolor*, **b** *P. chrysosporium*, **c** *P. sajor*-*caju*, **d** *P. ostreatus*. Note the difference in *Y-axis* scale

The extracellular enzyme activities are shown in Fig. 4. Laccase, MnP, and LiP were measured during the incubation period. None of the fungal strains showed LiP activity except for *P. chrysosporium* that showed low and not verifiable (large differences in repetitions) LiP activities, on days 6 and 9 of the incubation, although for the rest of the incubation period no significant LiP activity was detected. This may be due to the several reasons that have been suggested previously, such as inhibition of LiP by humic compounds and certain difficulties of the assay method (Lackner et al. 1991; Ralph and Catcheside 1994; Hofrichter and Fritsche 1997), inhibition of LiP activity of WRF due to the agitation (shaking) of the culture (Moyson and Verachtert 1993; Zanirun et al. 2009) or the absence of veratryI alcohol in the culture media used in this study, since it can induce and mediate the LiP activity (Waldner et al. 1988; Wesenberg 2003). The lack of LiP activity in the culture of the specific strain of *P. chrysosporium* that we used (DSMZ 1556), is not unprecedented (Blondeau 1989). Therefore LiP activity was excluded from the graphs in Fig. 4.

The measured MnP activity was much lower in the media containing HA compared to the control jars (media without HA). It has been reported before that the presence of HA in the media, interfere with the measurement of MnP, and results in underestimation of MnP activity (Ralph and Catcheside 1994; Hofrichter and Fritsche 1997). Therefore, it is not realistic to compare the level of MnP activities between fungal species in the presence of HA. However, the MnP activity in HA-free controls could be used qualitatively to prove the ability of fungal strains to produce MnP. When comparing the results of the laccase activity in media with and without HA in Fig. 4, it can be seen that in the presence of HA higher laccase activity was detected. The results suggest that HA could induce the laccase activity of WRF, which is in agreement with previous studies (Steffen et al. 2002; Kabe et al. 2005), and in contrast with some reports on inhibition of laccase by HA (Zavarzina et al. 2004).

*Phanerochaete chrysosporium* did not show any enzyme activity in the NS media during the first week but then started to produce MnP at a high rate. It is known that the MnP production by *P. chrysosporium* is part of a secondary metabolism that is triggered by scarcity in nutrients, namely nitrogen (Tien and Kirk 1984; Hataka 2005). The difference in the MnP production by *P. chrysosporium* could be explained by secondary metabolism conditions. *P. chrysosporium* in NL media enter the secondary mechanism conditions sooner than in NS media, so the MnP activity was detected sooner. In the second week, when *P. chrysosporium* enters the secondary metabolism phase in NS media, higher fungal biomass concentration in NS media results in higher MnP activity compared to NL media. For *Pleurotus* species, it seems that the production of extracellular enzymes was not part of a secondary metabolism, since enzyme activity was always higher when growing in NS media. *T. versicolor* showed high extracellular enzyme activities both in NL and NS media. It is known that *T. versicolor* can produce the extracellular enzymes both under limited nitrogen concentration (as secondary metabolites), and also in the presence of high nitrogen concentration (Bergbauer et al. 1991; Eggert et al. 1996).

*Trametes versicolor* showed higher laccase and MnP activity when growing in NS media, but showed more humic removal in NL media. When comparing the final concentration of HA after recovery in NL and NS media of *T. versicolor*, the HA concentration is higher in NS media, suggesting higher HA degradation in NL media, regardless of the higher enzyme activity in NS media. *P. chrysosporium* did not show any significant laccase activity, although it showed high MnP activity especially after 12 days of incubation in NS media. When comparing Figs. 3 and 4, for *P. chrysosporium*, it seems that the humic acid removal correlates with the MnP activity. Also, the SEC results after the recovery of sorped humics show higher recovery of large HA molecules (blue) in NL media than in NS media, suggesting higher degradation of HA in NS media. This correlates with the higher enzyme activity of *P. chrysosporium* growing in NS media.

**Fig. 5:**
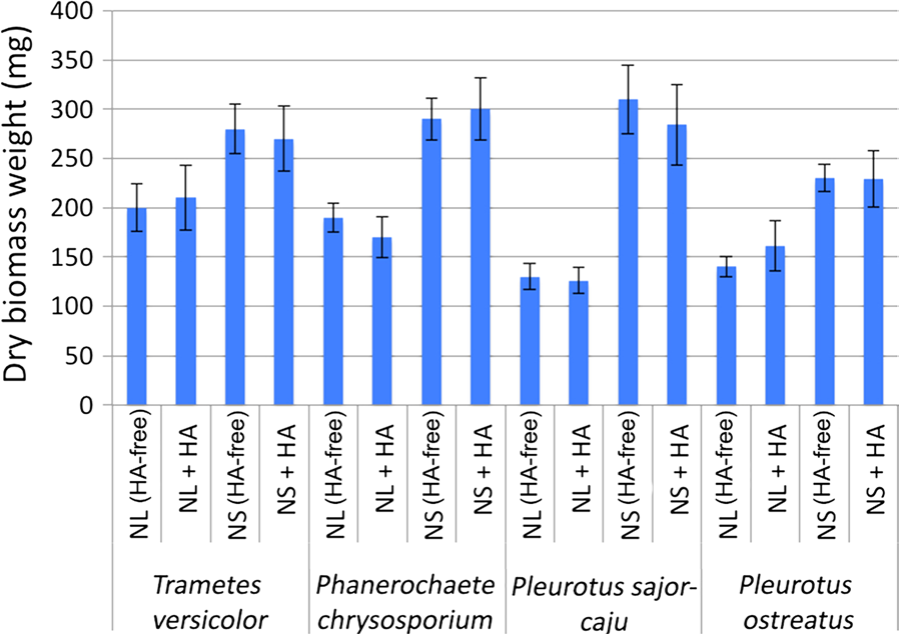
Weight of dry biomass produced after 18 days for four WRF strains

In order to study the effect of HA on the growth of WRF, the growth of WRF biomass was measured with and without the presence of HA. The results are shown in Fig. 5 as dry biomass weight (DBW) at the end of the incubation period.

The presence of humic acid in some cases enhanced the fungal growth and in some cases hindered the growth, especially for *P. sajor*-*caju*. These results can be compared with previous studies on the effect of humic and humic-like compounds on WRF’s growth (Klein et al. 2014; Mäkelä et al. 2015). When comparing the results of DBW in Fig. 5 with the results of humic removal in Fig. 3, there is no clear correlation between the increase or decrease of fungal biomass and humic removal. *T. versicolor* produced less biomass in NL media than in NS media (Fig. 5), but it removed more humics from water (Fig. 3). This suggests that biosorption is not necessarily the main mechanism of humic acid removal by WRF.

### Biosorption of HA by deactivated fungi

**Fig. 6:**
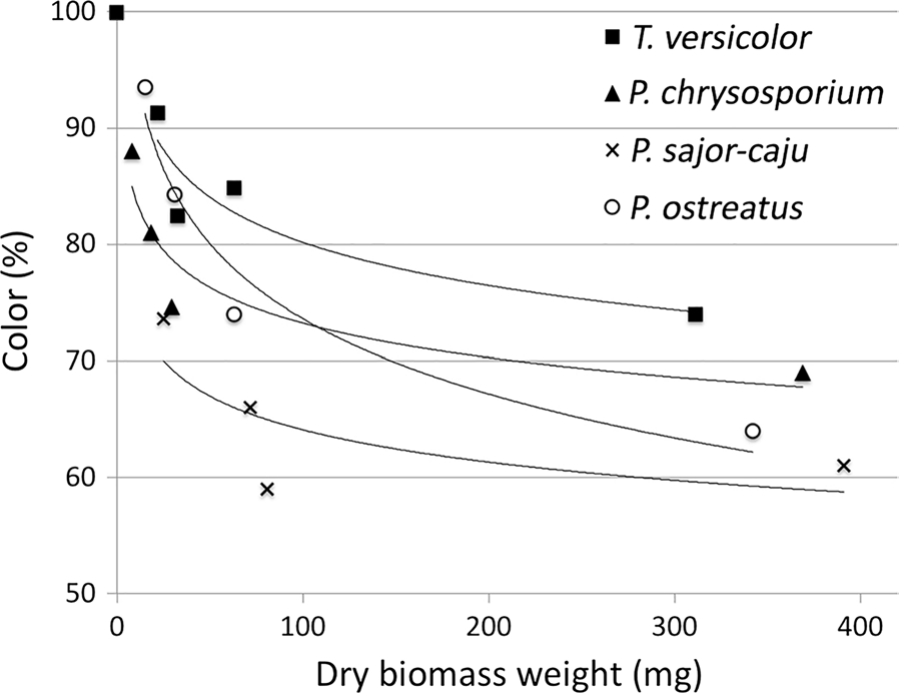
Biosorption efficiency as color removal by four different deactivated WRF

The biosorption of HA by deactivated WRF (shown by the decrease in color) is apparent from the results shown in Fig. 6, and it seems to be dependent on fungal species as well as the amount of biomass as sorbent.

*Pleurotus sajor*-*caju* showed the highest affinity for the biosorption of HA and *T. versicolor* showed the lowest affinity. The biosorption of HA (showed by the decrease in color) increased by increasing the fungal mycelia, although it showed a logarithmic trend, meaning that by increasing the fungal biomass the effect of the amount of biomass on biosorption of HA decreased. *T. versicolor* showed a maximum of about 25% biosorption with about 300 mg of biomass and *P. sajor*-*caju* showed about 40% biosorption with 400 mg of mycelia.

**Fig. 7:**
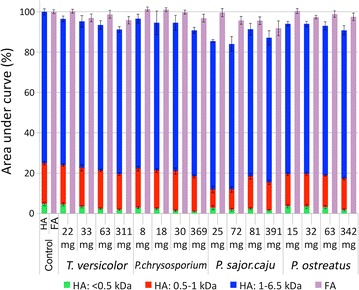
SEC results of the humic acid and fulvic acid portion of the humic compounds recovered from deactivated fungal mycelia after 48 h

The other important parameter to consider, was the efficiency of the HA recovery process, to see what fraction of the humics could be recovered (desorped) from the fungal mycelia. Results are shown in Fig. 7.

As it can be seen in Fig. 7, the efficiency of the humic recovery slightly varied among the fungal strains; it was not 100% efficient, and it decreased with the increase in fungal biomass. Nevertheless, the average efficiency of the recovery protocol was more than 85%. The deficiency of the recovery was mostly due to the smaller HA molecules (red and green) rather than the large HA molecules (blue). Almost in all cases large HA were fully (>96%) recovered from fungal mycelia, but in most cases, the smaller molecules (red and green) were not recovered completely, especially in case of *P. sajor*-*caju*.

Knowing the high efficiency of the recovery process, and taking the SEC results of the recovered HA in Fig. 3 into account, the role of enzymatic degradation of HA becomes more clear.

### Purified laccase

In order to study the effect of laccase on HA degradation, experiments were carried out using purified laccase in absence and presence of mediators.

**Fig. 8:**
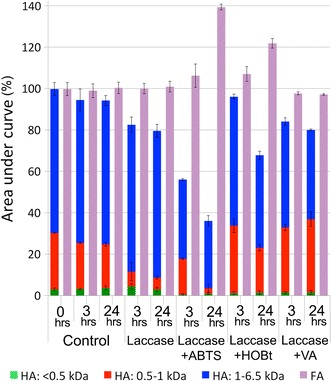
Degradation of HA by laccase, with and without the presence of mediators, expressed as SEC results (area under curve %) of HA and FA (Control: humic acid without enzyme and mediators)

It is clear from Fig. 8 that laccase can degrade HA. Although, when comparing the results after 3 and 24 h, it becomes apparent that the degradation is very slow when there is no mediator present. In the absence of mediators, only 20% reduction in the concentration of HA was observed after 24 h. ABTS proved to be the best mediator among the tested mediators. In the presence of ABTS, more than 60% of the humic acid was degraded after 24 h. The changes in the FA concentration was not as significant as in HA concentration. Although, in ABTS samples, a 40% increase in the FA concentration was observed. This simultaneous decrease in HA and increase in FA concentrations clearly shows the conversion of HA to FA by laccase in the presence of ABTS as the mediator. When using VA as the mediator, 20% of HA was degraded after 24 h, without significant change in FA concentration. Therefore it can be concluded that different mediators have different effects on the mechanism of HA degradation by laccase. The analysis of the MW distribution of HA confirms that the measurement of average MW is not necessarily representing the polymerization or depolymerization of HA. The concentration of HA was reduced to 36% of its initial value after 24 h of treatment with laccase in the presence of ABTS. This reduction is clearly due to the degradation of HA to non-aromatic compounds or conversion to FA-like substances, since there was no fungal biomass present, hence no biosorption could occur. However, the average MW of HA after 24 h was increased to 2 kDa, which implies polymerization of HA by laccase. The composition of the remaining HA in the media consisted of (after normalization to 100%) 90% large, 7% medium and 3% small HA molecules. In comparison with the initial composition of HA, shows a shift towards the larger molecules. The reason for this false implication is that when comparing the MW distribution and average MW of HA before and after the treatment, the non-aromatic products of the degradation of HA were not considered, since they cannot be detected via the UV detector during the SEC analysis.

### Role of cytochrome P450 enzymes

**Fig. 9:**
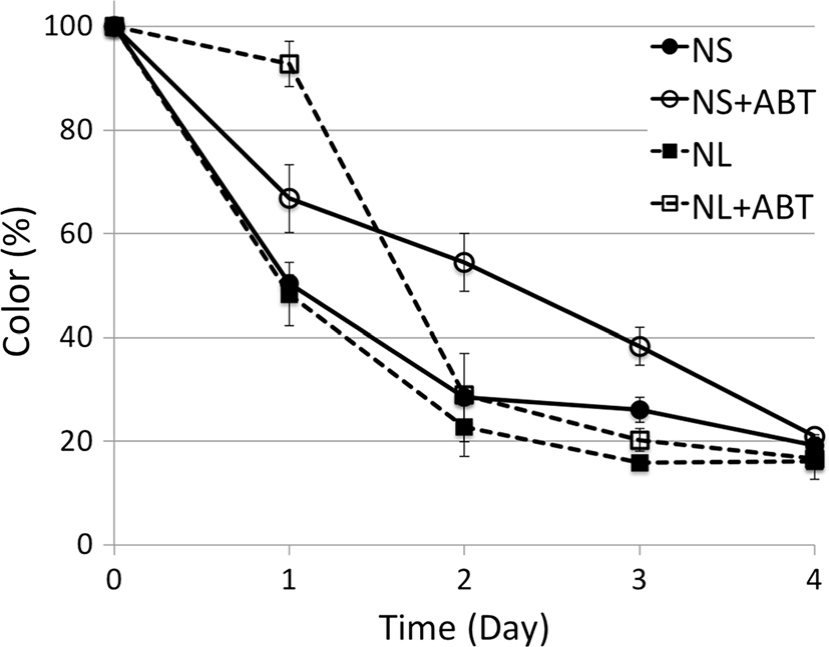
Role of CYP enzymes in HA bleaching by *T. versicolor*. ABT was used as the inhibitor of CYP enzymes

In order to investigate the involvement of the CYP enzymes in the degradation of humic acid, *T. versicolor* was incubated with and without the presence of CYP inhibitor. The results of the HA removal is presented as color removal in Fig. 9.

The presence of the CYP enzyme inhibitor decreased the rate of the humic acid removal significantly for the first 2 days. Although after 4 days, all samples reached almost 80% color removal. This observation suggests the contribution of CYP enzymes to the humic acid degradation. It seems that the CYP enzymes contribute most importantly to the rate of the degradation. It has been suggested that CYP enzymes mainly catalyze the reactions involving epoxidation of C=C double bonds and hydroxylation of aromatic compounds (Subramanian and Yadav 2008; Kelly and Kelly 2013).

## Discussion

The HA removal was assessed by measuring the decolorization of HA and confirmed by SEC analysis of the HA content of the media, and it was shown that the color (measured at 450 nm) correlated with the concentration of HA. Some previous studies used decolorization as a measurement of degradation of HA (Kabe et al. 2005; Grinhut et al. 2011b), based on research were decolorization of HA was observed when degradation of HA was also proven (Grinhut et al. 2011a). The co-occurrence of the decolorization and degradation of HA was also observed in our study; however, decolorization was not necessarily representing the degradation of HA. For example, in the case of *P. sajor*-*caju* (Fig. 3c), similar decolorization was measured at the end of the incubation period in the NL and NS media, but after desorption of HA, the SEC analysis revealed different degradation levels in the two media. Also, results of the HA removal by deactivated fungi proved that biosorption of HA (measured as decolorization) by fungi is not similar among fungal species. Therefore decolorization of HA is not necessarily representing the rate or extent of degradation.

In none of the experiments resulting in the degradation of HA, any increase in the color was observed. This is in line with previous reports on the decolorization of HA in the result of its degradation (Stevenson 1995) and in contrast with others reporting that the depolymerization of HA results in an increase in color (Zavarzina et al. 2004).

The measurement of average MW before and after the treatment was shown not to be a valid method to conclude on degradation and/or depolymerization or polymerization of HA. The reason is that the depolymerization and/or degradation of HA could basically result in three products, smaller HA molecules, smaller non-aromatic molecules, and FA. Except for the first group, the other products from the degradation of HA could not be detected in the SEC analysis (at 254 nm), and are therefore not considered in the calculation of the average MW.

These findings could clarify some confusion in the degradation (Steffen et al. 2002) or polymerization of HA (Zavarzina et al. 2004) by laccase. Looking closely at the method used in the polymerization studies, the average MW or MW distribution measured with SEC has been used for these claims, without considering the total area under the SEC curve which links to the concentration.

It was shown that different WRF species have different capabilities for biosorption of HA (Fig. 6). This might be due to the different structural properties of different fungal mycelia. The mechanism of biosorption of humic compounds by fungal biomass has been studied elsewhere (Zhou 1992; Urík et al. 2014). Also, It was shown that the large HA molecules were being recovered almost completely. Therefore any significant difference between the concentrations of large HA molecules (blue zone) at the beginning and the end of incubation (after the recovery), could be interpreted as degradation or conversion of these molecules. The observations of this study cannot necessarily prove the mineralization of HA by WRF, i.e. complete degradation to CO_2_ and water. However, it has been shown before that the degradation of HA by WRF enzymes could be associated with mineralization of HA molecules (Paper et al. 1998; Steffen et al. 2000).

From the results presented in this study, there is no clear and general correlation between extracellular enzyme activity (Fig. 4) and HA degradation (Fig. 3), which is in line with previous reports (Gramss et al. 1999). This might be due to the involvement of other extracellular enzymes such as versatile peroxidases (Siddiqui et al. 2014).
